# ‘*I will rather be killed by corona than by him…’*: Experiences of abused women seeking shelter during South Africa’s COVID-19 lockdown

**DOI:** 10.1371/journal.pone.0259275

**Published:** 2021-10-28

**Authors:** Bianca Dekel, Naeemah Abrahams

**Affiliations:** Gender and Health Research Unit, The South African Medical Research Council, Cape Town, South African; Universidad Pública de Navarra, SPAIN

## Abstract

**Background:**

In April 2020, the United Nations predicted that the COVID-19 pandemic will have a ‘calamitous’ impact on the lives of women. This was based on concerns about an upsurge in Intimate Partner Violence (IPV) arising from increased opportunities for relational conflict due to forced co-existence and therefore additional time spent with abusive partners.

**Aim:**

Research has shown an increase in IPV during times of crisis. The COVID-19 pandemic has generated unprecedented circumstances and stress, and opportunities to do research to understand whether the COVID-19 pandemic impacted on IPV experiences were limited. Thus, the present study aimed to understand women’s experiences of being in and leaving an abusive relationship during the COVID-19 pandemic.

**Methods:**

Individual, telephonic interviews were conducted with 16 women living in domestic violence shelters within three Provinces during South Africa’s lockdown period.

**Results:**

Findings reveal that the public health measures implemented by the South African Government to curb the spread of the virus, may have placed vulnerable groups at increased risk of violence. Specifically, lockdown likely magnified the risk for escalation of abuse in families already experiencing IPV prior to COVID-19. The study highlights an IPV and COVID-19 relationship, showing that the gender insensitive pandemic control measures, such as stay at home orders and travel restrictions, likely placed women at risk of increased abuse. Given the recurrency of COVID-19 epidemic waves, attention must be given to gender disparities or many South African women may experience worse outcomes.

**Conclusion:**

This study reminds us that being ordered to stay at home is not always the safest option for women and thus, in a country with one of the highest levels of GBV, it becomes imperative to ensure that IPV safeguards are integrated into COVID-19 measures. It also becomes evident that COVID-19 requires enhanced ways of responding by paying attention to gender disparities.

## Introduction

In 2020, the World Health Organization (WHO) declared the novel 2019-nCoV (Coronavirus Disease 2019) a global pandemic [1, 2]. Parallel to this visible COVID-19 pandemic exists a shadow pandemic of Gender-Based Violence (GBV) [3]. As early as 2014, UN Women used the term ‘pandemic’ to encapsulate the global pervasiveness of GBV [4]. Thus, there are two global pandemics occurring simultaneously where the one (GBV pandemic) is exacerbated by the other (COVID-19 pandemic) [3]. Early reports from countries first affected by COVID-19 (e.g., China), warned the rest of the world that these pandemics not only co-exist but also interact [5]. From the global start of the implementation of pandemic control measures, Intimate Partner Violence (IPV) has largely increased in a shadow pandemic [6]. Administrative and reporting data on GBV is not readily available and communicated within South Africa. Reliable service level data is yet to emerge on possible GBV increases during the COVID-19 period. However, many reports from newspapers and GBV service providers (NGOs) have reported an increase in GBV cases [7–10]. The head of South African Police Services released a media statement reporting on a general decrease in violent crime overall in the country for the 1^st^ seven days of the lockdown but warned that the same was not found for GBV, with more than 2300 calls registered in this period. As a comparison, the same report indicated that the number of GBV calls (received through the GBV Command Centre, a National, 24hr/7days-a-week Call Centre facility, operating under The Department of Social Development) registered per day during the first 90 days of the year (1 January– 31 March 2020) was 177 calls per day, which is a clear indication of the increased need for assistance sought by women in distress related to GBV during the lockdown [9]. This is not surprising given that South Africa is known to have high levels of GBV. For example, the country’s intimate partner femicide rates are amongst the highest in the world [11].

Similarly, reports from China, the United States of America, and several European countries have also shown an increase in IPV during the COVID-19 pandemic [12–16]. Thus, although pandemic response measures are essential, they do not adequately integrate gender protections to address morbidity and mortality from GBV (i.e., there is a lack of gender-sensitive pandemic control measures) [17].

For example, in South Africa, restrictive measures were taken, and a state of disaster was announced alongside a five phased lockdown approach (refer to Table 1 for more information), which was adopted and implemented on 27 March 2020. This lockdown severely restricted movement and limited people to only leaving home for the purchasing of essential items such as food and medical supplies [18]. The South African lockdown is considered as amongst the most restrictive globally: non-essential shops, businesses and restaurants were temporarily closed, schools were closed, all social and sport activities were banned, and there was a complete ban on the sale of alcohol and cigarettes [19]. In addition, President Cyril Ramaphosa deployed the army to assist the South African Police Services to ensure adherence to the COVID-19 regulations, specifically the restriction of movement [20]. Therefore, whilst the lockdown is arguably crucial, the restrictions implemented to reduce transmission as well as the pressure on the health care system, likely resulted in unanticipated consequences for women, as a few factors connect IPV and the COVID-19 pandemic [21].

**Table 1:** South Africa’s COVID-19 lockdown levels, period and summary of restrictions.

Lockdown level	Criteria for determination of alert levels	Period	Restrictions	Quarantine at shelters required
5	Indicates a high Covid-19 spread with a low health system readiness	26 March to 30 April 2020	Every person is confined to his/her place of residence, unless strictly for the purpose of performing an essential service, obtaining an essential good/service, collecting a social grant, pension or seeking emergency medical attention. Only essential service sectors allowed to operate. Essential employees commuting to work require a permit. Alcohol and cigarettes banned. All transport (cars and taxis included) to operate at restricted times of the day with limited capacity. No inter-provincial travel allowed.	Yes
4	Indicates a moderate to high Covid-19 spread with a low to moderate health system readiness	1 May to 31 May 2020	Food and retail stores permitted to be open and sell full line of products. Essential employees commuting to work still require a permit. Alcohol and cigarettes still banned. Waste services resume. Transport services may operate at all times of the day, with capacity limitations. Every person confined to his/her place of residence from 8pm– 5am, except for emergencies. Walking outside permitted between 6am-9am, within a 5-kilometer radius of place of residence. No inter-provincial travel.	Yes
3	Indicates a moderate Covid-19 spread with a moderate health system readiness	1 June to 17 August 2020	Take away restaurants and online food delivered. Alcohol sold within restricted hours. Cigarettes still banned. Transport may operate at all times of the day. Exercise permitted between 06:00 to 18:00.	Yes
2	Indicates a moderate Covid-19 spread with a high health system readiness	18 August 2020 to 20 September	Every person is confined to his/her place of residence from 22:00 until 04:00. Domestic workers allowed to return to work. Domestic air travel restored. Some provincial movement allowed, within Covid-19 restrictions.	Yes
1	Indicates a low Covid-19 spread with a high health system readiness.	21 September 2020—current	All sectors to resume within Covid-19 restrictions.	Yes

South Africa’s legacy of multiple inequalities may also arguably generate a setting ripe for the pandemic restrictions to exacerbate IPV. Thus, managing the relationship between COVID-19 and IPV, requires a careful response to the context and the intersectionalities that magnify the harmful impact of the pandemic restrictions among women in South Africa [17]. The primary lockdown requirement is confinement to the home, which minimizes opportunities for women to report violence and leave abusers [21]. Home isolation orders for women in abusive relationships often means being trapped (frequently without the means to access support) with a violent perpetrator who may become even more abusive as alternative outlets are minimized, such as social activities. Quarantine also presents abusers with an increased opportunity to inflict harm as they know that women and children have significantly reduced access to their support networks [22]. In fact, the COVID-19 lockdown is subjecting abused women to a compounded, veiled and unspoken lockdown as such women tend to already be exposed to controlling behaviours, which involve, for example, the prevention of seeing friends and family; while some women are not allowed to work, or to leave the house, suffering the risk of being abused if they do [23]. Indeed, lockdown is a crucial aspect of the COVID-19 public health emergency response, yet it magnifies the risk for increased IPV [21], which may further challenge an already fragile South African health care system.

Earlier literature has revealed that factors such as parenting stress [24], economic hardship [25], and food insecurity [26], increase the risk of violence, such as IPV. Indeed, many South Africans are experiencing food shortages and financial hardship during the pandemic as a result of job loss or reduced hours worked, which can worsen already stressful household situations. Simultaneously, we are reminded that many abused women tend to already be financially dependent on their abusers, making an attempt to leave quite difficult [19, 27]. There is also the presence of children and adolescents at home due to school closures, which may mean added parental stress [22]. Add these aforementioned factors, and the stress is intensified [28]. These factors may contribute to a perfect storm, a perfect recipe for perpetration and vulnerability [29].

However, to date, there is an absence of research that seeks to understand women’s experiences of being in abusive relationships during the COVID-19 pandemic. Thus, given that globally, we know little about the virus and its impacts, it is crucial that research is done to begin to understand the extent and depth of the impact that the COVID-19 restrictions have had on IPV in South Africa.

### Aim of the study

Based on the little that is known at present about how the pandemic restrictions may be impacting women in abusive relationships, we conducted an exploratory qualitative study to better understand such women’s experiences of being in and leaving an abusive relationship during the COVID-19 pandemic.

## Methods

A sample of 16 women (see Table 2) were recruited using purposive sampling. These 16 women were recruited from five shelters, based within both rural and urban areas within three South African Provinces. Six women were recruited from Western Cape shelters, six from a Gauteng shelter, and four women from a Kwa-Zulu Natal shelter. These shelters were selected as we wanted a racially diverse sample as well as women geographically dispersed within South Africa, and from both urban and rural areas. All five shelters provide residential shelter and refuge to abused woman and their children, along with counselling and skills training, in order to find healing, and to learn skills that will empower them to cope as self-reliant members of society. Recruitment occurred through each shelter’s social worker. The social workers spoke to each woman who met the inclusion criteria and asked each woman whether she would be willing to participate. If she agreed, a suitable date and time would be organized for the telephonic interview.

**Table 2:** Participant characteristics.

Participant name	Participant age	Race	Region	Entered shelter during level 5 COVID-19 lockdown
Tony	21	Black African	Gauteng	Yes
Caroline	44	Coloured	Western Cape	Yes
Ursula	20	Black African	Kwa-Zulu Natal	Yes
Belinda	44	Coloured	Gauteng	Yes
Michelle	46	White	Western Cape	Yes
Marsha	39	Coloured	Gauteng	Yes
Lauren	33	Coloured	Western Cape	Yes
Angel	52	Coloured	Western Cape	Yes
Sally	36	White	Kwa-Zulu Natal	Yes
Elizabeth	30	Coloured	Gauteng	Yes
Megan	24	Coloured	Kwa-Zulu Natal	Yes
Lucy	34	Coloured	Gauteng	Yes
Anna	34	White	Kwa-Zulu Natal	Yes
Lana	25	Black African	Western Cape	Yes
Unity	41	Coloured	Western Cape	Yes
Ingrid	32	Black African	Gauteng	Yes

Further, telephonic interviews (due to COVID-19 restrictions) were conducted in English in June 2020 with women housed at shelters for abused women and their children. The inclusion criteria was: Women (over the age of 18 at the time of interviews); who have experienced IPV from a male partner. Also, the abusive male partner could either be the participants’ former or current boyfriend/husband.

Throughout the data collection process, the first author engaged in reflexivity. This entailed writing notes to keep a record of thoughts, ideas, and observations. It was also a way to articulate beliefs and judgements, which was important to reflect on in terms of how these may have influenced the research process, in order to promote validity [30].

The first author conducted individual, semi-structured interviews, and each interview was on average, one hour in length. Repeat interviews were done with participants as two interviews were conducted per participant, which was based on a scope of enquiry, which was developed based on the literature reviewed, and used to guide the interviews. This allowed the agenda to be flexible although partially directed by the interview schedule. The first interview briefly explored their background and demographic information and then moved into the beginning of their intimate relationships. The second interview delved deeper into their relationships with partners, and also explored the eventual leaving of the abusive relationship.

Ethical approval was obtained from the South African Medical Research Council’s Human Research Ethics Committee. Approval to conduct research within each shelter was provided prior to the interviews by the Social Worker at each organization. Written informed consent procedures were followed and the aim of the study as well as all ethical procedures (risks and benefits, anonymity, confidentiality) and the recording of the interviews were explained to participants. To maintain anonymity, we use pseudonyms in the paper. We recognized that support for participants was vital and thus, prior to each interview we asked each social worker whether he/she would be willing to meet with the women after the interview, if she felt this was needed. This would have been followed up by the first author to ensure that all participants requesting this service, received it, however, no participants requested additional therapy. Psychological support was also arranged for the first author, who conducted the interviews. After the interviews were conducted, the first author transcribed some of the interviews and a transcriber assisted with the remaining interviews. All interviews were transcribed verbatim. Quality checks were done by the first author who conducted crosschecking of all the recorded interviews against the transcribed interviews.

Data analysis was performed according to the principles of grounded theory. During open coding, audio-recordings and interview transcripts were reviewed with the aim of examining the text for thoughts, ideas, and meaning and consequently assigning them codes, and consequently a codebook was developed. Emphasis was placed on allowing concepts to emerge naturally without forcing them into predefined categories. Categories were then divided into sub-categories. Trimming down of the codes was done with the assistance of the second author, thereby concluding the first stage with 21 codes. Together, a single category was identified (axial coding) as the central phenomenon of interest. This selection was made based on the categories most extensively discussed by participants (i.e., the codes with the highest frequency), which were then positioned as central features, around which, other categories were related, and a storyline was constructed. To illustrate this storyline, discriminate sampling was used, which refers to selecting certain participant quotes, which are able to maximize opportunities for verifying the storyline. The storyline was further validated by searching for relevant literature pertaining to categories. Member checking was performed with participants to iron out ideas and reach consensus. Lastly, core categories were then organized into the research paper. Quotes used herein are illustrative of the codes selected.

### Findings

The study includes 16 participants, racially categorized as African (4), Colored (9) and White (3) (using the Apartheid racial classification system). Despite women coming from different racial groups, they had similar demographic profiles, living in communities where socio economic hardships were common, with all the women reporting financial difficulties, which predated the pandemic. Women described their communities as plagued by violence and crime, poor policing, gangs, unemployment, alcoholism, drug addiction, and domestic violence.

#### Women’s experiences of Intimate partner violence during South Africa’s COVID-19 lockdown period

Most of the women tearfully explained how tough it was to be forced (as per Government regulations) to self-isolate with their abusers, as Tony (aged 21 from Gauteng) explains, ‘*It was horrible*. *I can’t even explain*. *Like this man couldn’t go to work or anywhere and also me I couldn’t*. *So*, *we were both stuck at home and we didn’t want to be there every day*, *day in and day out*. *We were on top of each other*. *Every little thing I did made him mad*. *I have never been so stressed in my life*. *I felt like I was gonna die from stress*’. Tony’s excerpt provides a glimpse into the notion that a one size fits all approach is not the best. For example, the response to lockdown South Africa, because it worked in China [31, 32] does not take into account South Africa’s idiosyncrasies, and does not responsibly address the country’s social and economic landscape [33], specifically South Africa’s high IPV rate [34–36].

Currently, many South Africans live in overcrowded conditions with homes in informal dwellings, accommodating large or multiple families. Thus, maintaining social distancing or quarantining the infected is almost impossible. In fact, confining such families to the home may further contribute to disease transmission, especially during winter as people tend to crowd into poorly ventilated spaces [33]. Thus, while COVID-19 is global in its impact, tackling the pandemic requires a response that is tailored to the local context [33].

Further, most participants explained that the Government imposed COVID-19 restrictions removed their ‘usual’ escape strategies. Unity (aged 41, residing in the Western Cape Province) explains, ‘*Before the lockdown*, *after he beat me*, *I could go outside and take a walk and cry and come back when I stop crying*. *Or if I see he is gonna beat me*, *I can maybe quickly run outside and hide in the bush and come back when he’s sleeping*. *But with the lockdown*, *oooh no there was none of that*. *You must stay inside and take each and every one of your beatings*. *You can’t even escape one beating*’.

Alongside the removal of typical escape strategies, was the worsening of the abuse, which was largely the reason why these women decided to leave their abusive relationships, as the abuse they were experiencing escalated into near femicides for most, which is outlined in a separate publication. The participants in this study recognised that the abuse was exacerbated by stressors related to the pandemic and that the frequency or severity of the abuse was not declining [37].

All of the women were already experiencing abuse before the emergence of COVID-19, as Ingrid (aged 32 from Gauteng) explains, ‘*Yor he used to beat me before corona*, *while I was still pregnant*. *You know*, *he dragged me from the lounge to the bedroom by my hair while I was pregnant with a big stomach*’. However, they all expressed that the abuse worsened during the lockdown period. For example, Sally (aged 36, from Kwa-Zulu Natal) stated that, ‘*Before corona*, *he used to beat me and that’s it and I will get up and go and wash myself or whatever*. *I noticed during the corona lockdown that the beatings definitely got worse*. *During corona*, *it was the first time that I really thought to myself*, *‘geesh*, *he is really going to kill me’*. *It is the first time the idea of being killed entered my mind*. *Like*, *during the corona lockdown*, *it was the first time he beat me and then after that went to fetch a rope and tried to hang me*’. Likewise, all the women shared Megan’s (aged 24 from Kwa-Zulu Natal) sentiments that, ‘*During the lockdown*, *the abuse was getting worse’*.

#### Increased levels of stress

*Economic stress*, *parenting responsibilities and restrictions*. The women reported that during South Africa’s hard lockdown period, the abuse worsened, largely because of increased stress within the home. They mentioned the contributing COVID-19 related factors which included job loss/loss of income, increased parenting responsibilities, and less access to alcohol and cigarettes. These factors served to increase the risk of IPV, while making a potential escape less feasible.

Megan, who stated that the abuse was, ‘*getting worse’* during the lockdown, sheds light as to why the abuse worsened, *‘I think it is because we didn’t have money*… *with the lockdown I couldn’t work’*. Indeed, due to prior conditions and the added burden of the COVID-19 national response, there has been an increase in levels of poverty and malnutrition [38]. Some wealthier South Africans may be able to depend on continued salaries or savings to get them through the lockdown. However, for the less privileged, there are often no reserves and many South Africans have lost their day-to-day source of livelihood [33].

Megan further elaborates, *‘So*, *there was no money and no money now for his drugs’*. Indeed, some men who lack financial resources may resort to violence to try to re-establish power within the home [39].

Megan’s narrative highlights the multiple COVID-19 related stressors that come to the fore and provides a glimpse into onto how the COVID-19 restrictions exacerbated her partners’ stress, *‘Oh my word and plus he couldn’t buy cigarettes and alcohol*, *remember*? *So*, *he was very cross*. *Also*, *my kids couldn’t go to school during the lockdown and now they were at home and bored and making a noise whole day and he was just getting cross every day*. *He was getting crosser and crosser every day and I was getting stressed every day’*. Furthermore, the adverse economic situation brought on by COVID-19 meant women were trapped, as outlined by Megan, as she explained her stress increased as she was not able to escape a very angry partner, ‘*I didn’t evens have money for transport to leave him*. *Also*, *now if I leave him*, *do I take my kids with*? *Then what if they get corona*? *Or do I leave them with him*, *but who will look after them*? *I had all such thoughts*. *It was very hard*’.

Similarly, Elizabeth (aged 30 from Gauteng) also explained that for the first time, her children went hungry, ‘*With the virus*, *there was no work*, *it was the most difficult time of my life*. *This was the first time ever I was without work*. *Before lockdown*, *my children has never skipped supper*, *even if it was only a egg with two slices of bread*, *but during lockdown*, *there was times my children ate nothing*. *It was just too much*. *I can’t explain that stress to you*. *You know what made it worse*? *My husband never cared*. *He would eat the last bit of rice*, *even though it was not his*. *I would go and beg for some rice to give the children and then he would eat it*. *When I looked to give it to the children*, *there was nothing*’. Elizabeth’s excerpt provides a glimpse into how the Governmental response to the pandemic exacerbated adverse conditions as many families faced and are still currently facing, economic crises as a result of some pandemic response policies.

*Lack of social support and travel restrictions*. In addition to these factors, Governmental orders to stay at home, prohibited the visiting of friends and family, which removed social support, as Elizabeth (aged 30 from Gauteng) explains, ‘*I had nobody*, *no family*, *you understand… I was alone in the house with an abusive man and my children*. *It was very tough*’.

Moreover, the South African Government imposed travel restrictions greatly limited mobility and therefore diminished the likelihood of leaving the relationship. Michelle (aged 46 from the Western Cape) explains, ‘*I don’t have a car and remember we needed permits to travel and I didn’t have one*. *I didn’t even know if taxis and busses were coming to my area*. *Remember the army was also out there*. *It was very scary*. *I felt like I was going to be arrested if I was walking so I felt so stuck*. *So so stuck*. *It made me so stressed and depressed*’.

In spite of the many challenges, all of the women were able to leave the abusive relationship. Some examples of how these women eventually escaped the relationship included, ‘*leaving my kids with the maid and I ran away with a bag of clothes*. *I hid next door and then the neighbour found me a shelter by googling for me and taking me there*. *It was so hard to leave my kids*, *but I had to*. *I couldn’t take it anymore*. *I had to save myself because he was threatening to kill me*’ (Sally) or ‘*There is a policewoman that lives in my road*. *She knows me well*. *She could see I was in an abusive relationship and the one time she did said to me if I ever need help*, *I must come to her*. *So*, *the one night I couldn’t take it anymore and I just ran to her house crying*. *So*, *she did phone her people at the shelter and then she did bring me here*’ (Megan).

#### Fear and threats for leaving: ‘*If you walk out that door*, *you gonna get corona and die’*

In addition to the Governmental imposed travel restrictions, some of the women reported that their male partners engaged in psychological abuse and used COVID-19 to instil fear in them in an attempt to prevent them from leaving, as Lana (aged 25 from the Western Cape) explains, ‘*He would tell me*, *‘if you walk out that door*, *you gonna get corona and die*.*’ He would tell me that every day*. *And what must I believe*? *Because they are saying on the news that it is so*, *so I was believing him*. *I wasn’t thinking he is trying to control me*, *you know*? *So*, *I stayed until I couldn’t anymore*. *I stayed until I thought okay let me rather walk out that door and get corona and die*, *then he can’t beat me anymore at least*’.

All of the women elaborated on the unique and distressing paradox they found themselves in, due to COVID-19: Either they could remain in the relationship, running the risk of enduring and/or escalating violence, which could lead to death, or they could leave the relationship and risk exposure to a highly infectious virus that could lead to death. Caroline (aged 44 from the Western Cape) explains, ‘*It was like*, *now do I try to escape from this abusive man*, *or do I go out there and get corona*? *Both options was very scary*. *Both options was so stressful*’. Caroline’s excerpt provides a glimpse into the mental anguish entailed in leaving an abusive relationship during South Africa’s lockdown, which created a huge dilemma that women who left an abusive relationship outside of lockdown, did not have to consider.

Likewise, Megan also reported that, ‘*It was very hectic*. *I was so afraid of leaving because I didn’t wanna be in quarantine*, *I was very scared*. *We were quarantined for fourteen days at the shelter and that was scary*. *Before I left*, *all I was thinking to be honest*, *was that it is very scary out there because of corona*. *It was just scary to be honest*, *but I did not let corona stop me*. *Eventually*, *I decided now is the time for me to do something about my life*, *coronavirus or not*, *now is the time*. *I just stopped caring; I was just too scared of my children’s father*’.

Thus, some of the women explained that their fear of their male partners outweighed their fear of contracting COVID-19, as Angel (aged 52 from the Western Cape) explains, ‘*It was very frightening to leave during corona*, *because of the fact of the virus itself*. *I am also a chronic patient*, *so I have higher risks*. *But my fear didn’t stop me from leaving*. *It didn’t frighten me enough to not leave the relationship*. *Because*, *the first thing that came into my mind was*, *I will die in this relationship*. *I could die out there of corona*, *but I would rather take my chances with that*. *I will rather be killed by corona than by him*, *because if I stay here*, *he will kill me*, *so I will rather take my chances with corona*’.

## Discussion

**Fig 1:**
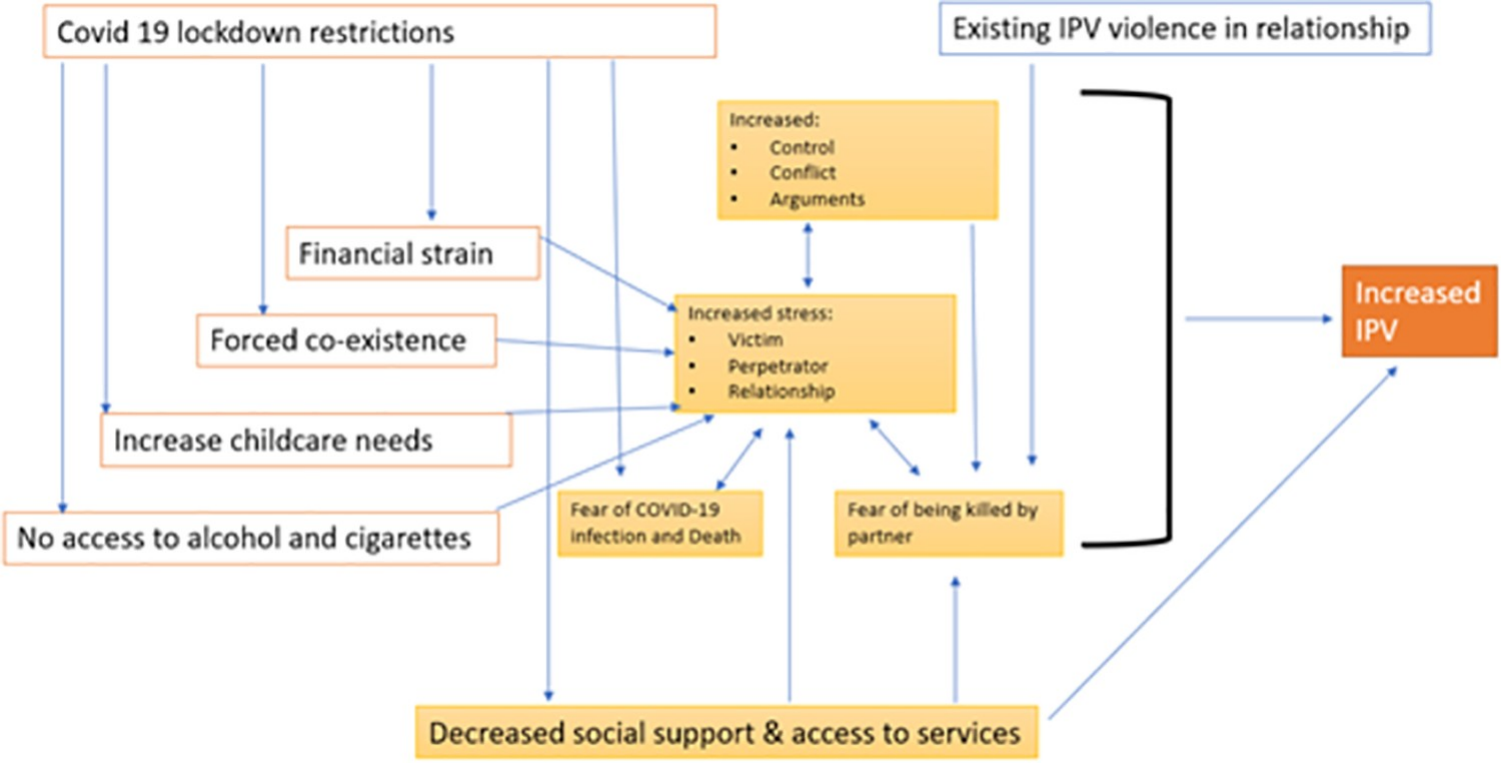
How COVID-19 restrictions influenced household dynamics and increased IPV.

Nationwide stay-at-home policies following the introduction of the COVID-19 pandemic, abruptly interrupted daily life across South Africa and the globe. In this paper we show how lockdown restrictions introduced new stressors and exacerbated existing strains, thereby escalating violence within already abusive relationships within South Africa (Fig 1). It is, however, worth bearing in mind that a limitation of this study is that the participants represent a specific group of abused women, who arguably have greater agency and access to resources, as they were able to leave their abusive partners during the pandemic. Thus, the voices of those women who were unable to leave their abusers, are not included. Future research is needed to understand IPV experiences amongst those women who were unable to leave during the pandemic.

Evidence of escalation and increase in severity of violence experienced by women was also reported in a study of trauma patients in South Africa, where all other forms of injury admissions decreased but female trauma admissions increased to levels higher than before lockdown [40]. Similarly, a recent study conducted by Jetelina, Knell, and Molsberry [41] in the USA found that 17% of female respondents reported that physical IPV worsened during the early stages of lockdown. This should not be surprising as pandemics tend to have accompanying risk factors such as, intense stress, financial difficulties, and decreased social support (as evidenced in this paper), which are also risk factors for IPV and therefore abuse in relationships may worsen during times of crises [17].

Indeed, due to the restrictions implemented during the COVID-19 lockdown period, this period emerged as an opportunity for abusers to tighten their controlling grip on their female partners. This study has shown that the fear and stress associated with pandemics may provide an enabling environment that may exacerbate IPV. Government regulations such as social distancing, inadvertently increased the risk of IPV. Travel restrictions could amplify this dynamic, as many South Africans found themselves in situations in which public transportation and travel by car were limited. Thus, this study has shown that within an isolated setting, some male partners used coercion and control to keep women at home during the lockdown period. Although, intimidation, manipulation, and control are long-standing hallmarks of abusive relationships [42, 43], this discovery is in line with preliminary findings from the USA where concerns have been raised that abusers have been utilizing COVID-19 to instil fear and compliance in female partners as a means of further isolating them [44, 45]. It is possible that this type of coercion might also have resulted in fewer victims reporting IPV and/or seeking medical care for IPV-related injuries [44, 46, 47].

Tied to this, is the paradox highlighted in this study, which many of the women faced, that is, to either stay in the abusive relationship at home thereby adhering to Government regulations to possibly avoid contracting the disease, yet running the risk of enduring or escalating violence; or to leave the home and risk exposure to the coronavirus. The women in this study chose the latter, despite facing a paradoxical risk of dying. Thus, there are two global pandemics occurring simultaneously where the one (GBV pandemic: less visible and efforts to address it significantly under-resourced and overlooked) is exacerbated by the other (COVID-19 pandemic: highly visible, receiving significant public attention and resources) [3].

Overall, this study has reminded us that being ordered to stay at home is not always the safest option for women and that pandemic response measures must include local vulnerabilities [48]. In fact, the home is often the space where abuse occurs as the home is frequently a place where power dynamics can be undermined by those who abuse, often without scrutiny from anyone ‘outside’ the couple, or the family, especially during lockdown restrictions where mobility is restricted and visits from friends and family are prohibited. Thus, during the COVID-19 pandemic, the order to ‘stay at home’ has huge and potentially, deadly consequences for women who are living with a violent partner. Strict movement restrictions minimize opportunities for escaping or for seeking help for victims. As this study has shown, these restrictive measures tend to play into the hands of people who abuse through tactics of control, manipulation and coercion. Unintentionally, these lockdown measures grant abusers greater freedom to act without scrutiny or consequence [49].

Therefore, in a country where 42% of men have reported IPV perpetration [34] and between 43% and 56% of women have experienced IPV [35, 36], it becomes imperative to think critically about orders to stay home. It is understood that it has been essential to alter social behaviours to stop the spread of the virus so as to save lives and to protect our fragile health care systems. However, this study raises concerns surrounding abused women as a way of highlighting some of the paradoxes of such measures. Thus, highlighting the absence of imperative gendered considerations and voices in South African pandemic control measures, which are facilitating a context where IPV tends to increase during the COVID-19 pandemic. Important to note is that we are not opposing such measures, but instead are bringing attention to the voices and needs of abused women, which tend to be slightly forgotten within some policy and political spheres, evident in the lack of gender-sensitive pandemic control measures.

### Implications

Pandemics tend to have damaging consequences for people, communities, and countries. However, this devastating impact is usually not uniform, as the impact is more severe in some groups. Thus, to adequately prevent and address the varying effects of pandemics, it is vital to bear in mind how our gendered differences lead to different pandemic experiences, which require tailored, gender sensitive pandemic control measures [50].

Indeed, there are various steps that Government can take to mitigate the IPV and COVID-19 connection. As a start, it is crucial that the South African Government ensures that public services for abused women are not only open but prioritized and integrated into COVID-19 programs [21]. Mitigating this relationship may also mean finding novel and creative ways to make IPV resources accessible to all, via multiple platforms. Since many South Africans live in poverty and are experiencing added financial difficulties due to the COVID-19 pandemic, some individuals may have limited access to IPV resources via television, smartphone, and the internet [51]. We are also reminded that prior to the pandemic, approximately only 39.7% of South Africans owned a smartphone [52]. Thus, paper flyers and the use of community radios providing information about IPV resources can be a form of communication within communities [51].

Indeed, the way in which help reaches individuals experiencing IPV may be different during a pandemic. There may be an increased and dire need for discreet ways to access IPV services. Home confinement orders may result in greater stalking and control, therefore, limiting women’s ability to call [53]. Thus, hotlines providing options to text or chat online are helpful. In South Africa, M-Health apps (some existing prior to the COVID-19 pandemic), such as the Gender Based Violence Command Centre, a 24/7 support service, and Bright Sky, a free app that provides support and information to those affected by IPV [51], are emerging as creative alternatives, yet its effectiveness has not been assessed. Social media can play an important role by raising awareness and providing a direct link to formal assistance/support as well as a link to friends and family during the lockdown, possibly mitigating the psychological effects of isolation, and allowing victims to reach out for help if needed [54]. Overall, the importance lays in diversified channels of interaction so that all abused women can access support [53].

However, some abusers might monitor their partner’s cell phone activity or withhold their phones from them. In this case, pandemic response measures need to allow women to leave the home. Thus, we encourage the Government to make exceptions for individuals experiencing IPV to leave home to seek help while under lockdown or stay-at-home orders. For example, in order to adhere to COVID-19 guidelines, mobile health clinics may be implemented within communities as these largely increase physical accessibility and may decrease possible exposure to COVID-19 due to, for instance, not necessarily having to travel by cramped, poorly ventilated transport options [17]. To accompany this, information about these exceptions should be widely publicized [51]. Alternatively, South Africa is also encouraged to follow those international countries, such as France and Spain, that have adopted code-word systems, which have been implemented in essential businesses, such as food stores, so that women may notify an employee that they are experiencing IPV and need assistance [54, 55]. The devastating nature of COVID-19 and its link to an upsurge in IPV globally, requires additional international and national research to be conducted. Such studies could build upon these findings by ascertaining the effectiveness of the proposed strategies for women in abusive relationships [53].

Furthermore, this paper has shown that the women in this study largely received assistance from those people in close proximity to them. Therefore, we encourage Government and policymakers to underscore the need for South Africans to keep in touch with each other (while observing precautionary measures) in order to maintain social connectedness, especially with family or friends who may be at risk of violence. In addition, information about locally available services, such as shelters, should be made known to the public so that everyone is in a position to connect those in need with the relevant support services, if need be [56].

Going forth, although Governments implement measures to tackle COVID-19, there remains a need to extend these efforts to specifically address the IPV and COVID-19 relationship. IPV survivors as well as members from local women’s organisations, should be included in developing response measures related to catastrophic events [17, 53] as they are arguably best situated to design measures that can be implemented in a way to mitigate IPV risks [17]. This is important as there are arguably gender disparities in preparedness, response, and recovery plans and there remains a need to ensure a stronger, gender-sensitive, catastrophic/emergency response [50]. This is especially vital as lifesaving GBV services become vulnerable to cessation when the effort and focus is to prevent and control the spread of deadly diseases, without incorporating a gender-sensitive lens. Thus, it is not the COVID-19 disease that tends to increase the risk of IPV, but rather the gender-insensitive policies and systems that increase the risk. Decision makers should be cautioned against believing that lives saved from COVID-19, carries more importance than the lives impacted by and/or saved from IPV [17].

## Conclusion

The South African Government has requested all South Africans to contribute towards fighting this pandemic, yet a critical reflection as to what this entails for many women is key. This is especially important as we prepare for multiple waves of the virus and thus, there needs to be a strong effort to ensure IPV safeguards are integrated into COVID-19 measures [57]. The United Nations Secretary-General highlighted the need to integrate protections for women during COVID-19. Specifically, all COVID-19 response plans need to address the impact of this pandemic on women and placing women at the heart of the response effort will ensure better and more sustainable development outcomes and support a faster recovery. Thus, given South Africa is a member state, this is a call to action for the South African Government [58]. In sum, this paper attempts to expose that which has been made invisible and render audible what has been silenced—the abused women’s experiences under COVID-19—in order to assist all women during the COVID-19 and any other emerging pandemics.

## Supporting information

S1 File Participant quotes. (DOCX)
