# Supplementary material for: ‘I will rather be killed by corona than by him…’: Experiences of abused women seeking shelter during South Africa’s COVID-19 lockdown
Source: PLoS One. 2021 Oct 28;16(10):e0259275. doi: 10.1371/journal.pone.0259275 (PMC8553161; doi:10.1371/journal.pone.0259275)
Supplement: S1 File — (DOCX) [file pone.0259275.s001.docx]

**Megan:**

‘*During the lockdown, the abuse was getting worse’.*

*‘I think it is because we didn’t have money... with the lockdown I couldn’t work’.*

*‘So, there was no money and no money now for his drugs’.*

*‘Oh my word and plus he couldn’t buy cigarettes and alcohol, remember? So, he was very cross. Also, my kids couldn’t go to school during the lockdown and now they were at home and bored and making a noise whole day and he was just getting cross every day. He was getting crosser and crosser every day and I was getting stressed every day’.*

‘*I didn’t evens have money for transport to leave him. Also, now if I leave him, do I take my kids with? Then what if they get corona? Or do I leave them with him, but who will look after them? I had all such thoughts. It was very hard*’.

‘*There is a policewoman that lives in my road. She knows me well. She could see I was in an abusive relationship and the one time she did said to me if I ever need help, I must come to her. So, the one night I couldn’t take it anymore and I just ran to her house crying. So, she did phone her people at the shelter and then she did bring me here*’.

‘*It was very hectic. I was so afraid of leaving because I didn’t wanna be in quarantine, I was very scared. We were quarantined for fourteen days at the shelter and that was scary. Before I left, all I was thinking to be honest, was that it is very scary out there because of corona. It was just scary to be honest, but I did not let corona stop me. Eventually, I decided now is the time for me to do something about my life, coronavirus or not, now is the time. I just stopped caring; I was just too scared of my children’s father*’.

**Tony:**

‘*It was horrible. I can’t even explain. Like this man couldn’t go to work or anywhere and also me I couldn’t. So, we were both stuck at home and we didn’t want to be there every day, day in and day out. We were on top of each other. Every little thing I did made him mad. I have never been so stressed in my life. I felt like I was gonna die from stress*’.

**Unity:**

‘*Before the lockdown, after he beat me, I could go outside and take a walk and cry and come back when I stop crying. Or if I see he is gonna beat me, I can maybe quickly run outside and hide in the bush and come back when he’s sleeping. But with the lockdown, oooh no there was none of that. You must stay inside and take each and every one of your beatings. You can’t even escape one beating*’.

**Ingrid:**

‘*Yor he used to beat me before corona, while I was still pregnant. You know, he dragged me from the lounge to the bedroom by my hair while I was pregnant with a big stomach*’.

**Sally:**

‘*Before corona, he used to beat me and that’s it and I will get up and go and wash myself or whatever. I noticed during the corona lockdown that the beatings definitely got worse. During corona, it was the first time that I really thought to myself, ‘geesh, he is really going to kill me’. It is the first time the idea of being killed entered my mind. Like, during the corona lockdown, it was the first time he beat me and then after that went to fetch a rope and tried to hang me*’.

‘*leaving my kids with the maid and I ran away with a bag of clothes. I hid next door and then the neighbour found me a shelter by googling for me and taking me there. It was so hard to leave my kids, but I had to. I couldn’t take it anymore. I had to save myself because he was threatening to kill me*’.

**Elizabeth:**

‘*With the virus, there was no work, it was the most difficult time of my life. This was the first time ever I was without work. Before lockdown, my children has never skipped supper, even if it was only a egg with two slices of bread, but during lockdown, there was times my children ate nothing. It was just too much. I can’t explain that stress to you. You know what made it worse? My husband never cared. He would eat the last bit of rice, even though it was not his. I would go and beg for some rice to give the children and then he would eat it. When I looked to give it to the children, there was nothing*’.

‘*I had nobody, no family, you understand… I was alone in the house with an abusive man and my children. It was very tough*’.

**Michelle:**

‘*I don’t have a car and remember we needed permits to travel and I didn’t have one. I didn’t even know if taxis and busses were coming to my area. Remember the army was also out there. It was very scary. I felt like I was going to be arrested if I was walking so I felt so stuck. So so stuck. It made me so stressed and depressed*’.

**Lana:**

‘*He would tell me, ‘if you walk out that door, you gonna get corona and die.’ He would tell me that every day. And what must I believe? Because they are saying on the news that it is so, so I was believing him. I wasn’t thinking he is trying to control me, you know? So, I stayed until I couldn’t anymore. I stayed until I thought okay let me rather walk out that door and get corona and die, then he can’t beat me anymore at least*’.

**Caroline:**

‘*It was like, now do I try to escape from this abusive man, or do I go out there and get corona? Both options was very scary. Both options was so stressful*’.

**Angel:**

‘*It was very frightening to leave during corona, because of the fact of the virus itself . I am also a chronic patient, so I have higher risks. But my fear didn’t stop me from leaving. It didn’t frighten me enough to not leave the relationship. Because, the first thing that came into my mind was, I will die in this relationship. I could die out there of corona, but I would rather take my chances with that.* *I will rather be killed by corona than by him, because if I stay here, he will kill me, so I will rather take my chances with corona*’.
